# The midwifery clinical faculty model: An innovation in midwifery education in Iran

**DOI:** 10.18332/ejm/210326

**Published:** 2025-09-30

**Authors:** Monireh Toosi, Azar Nematollahi, Zahra Rastegari, Parvin Yadollahi

**Affiliations:** 1 Community Base Psychiatric Care Research center, School of Nursing and Midwifery, Shiraz University of Medical Sciences, Shiraz, Iran; 2 Department of Midwifery, School of Nursing and Midwifery, Shiraz University of Medical Sciences, Shiraz, Iran; 3 Department of Midwifery, Maternal-fetal Medicine Research Center, School of Nursing and Midwifery, Shiraz University of Medical Sciences, Shiraz, Iran

**Keywords:** education, midwifery, midwifery students, clinical education

In Iran, midwifery students are admitted through a national examination over a four-year academic period. The midwifery education program has been designed by the Ministry of Health as a single curriculum for universities across the country. It includes theoretical and practical courses over a four-year midwifery period. Most midwifery courses are devoted to acquiring clinical skills, during which students work in groups of 4 to 8 in clinical settings. To complete the midwifery course, students must participate in at least 60 natural deliveries and must successfully pass the comprehensive midwifery exam to graduate. All undergraduate students must complete 142 credits in the form of 112 theoretical credits and 30 clinical credits over four years. After completing all of the aforementioned credits, they must take a final exam to graduate, and success in the exam is a prerequisite for graduation. Also, in Iran, graduates with a Bachelor’s degree in midwifery have the opportunity to pursue their education at a Master’s or Doctoral level in midwifery. Based on their missions, midwifery schools in Iran should train graduates who have sufficient capabilities in the prevention, treatment, and health promotion of mothers and infants. To have maximum efficiency, midwifery students should participate in theoretical classes, while also acquiring clinical skills through practice and gaining experience in clinical settings^1-6^. The midwifery clinical education in Iran is challenged by certain factors; due to the shortage of clinical professors possessing sufficient knowledge and skills for teaching as well as practical and specialized practice, educational managers have clinical personnel teach nursing and midwifery students by using traditional nursing and midwifery education methods^7^. Although most clinical preceptors possess useful clinical experience, for the purpose of education, those who adopt the role of clinical teachers should also possess new scientific information to teach theoretical topics. Still, due to their departure from academic education or because of time restrictions, some clinical personnel change the process of care, eliminate many standard care steps, and create so-called shortcuts in the accurate implementation of care^8^. However, to be educational, these cares should be performed in their complete and up-to-date form by the teacher. In the traditional midwifery education model, most students experience educational contrasts and confusion; on the one hand, theoretical topics are transferred to them in their academic form, and on the other hand, they encounter teachers in clinical settings whose performance runs contrary to those theoretical topics. In Iran, clinical settings such as hospitals are separate from academic settings, and midwifery professors are in fact guests in clinical settings and rarely wield administrative power^7^. These conditions cause conflicts between midwifery professors and the personnel, doctors, and obstetrician-gynecologists. To resolve these problems and integrate universities and clinical settings, measures such as the implementation of the midwifery clinical faculty model (MCFM) have been taken in Iran. In 2014, MCFM was notified by the Ministry of Health and Medical Education to the universities across the country, and was implemented in some universities, including Shiraz and Ahvaz Universities of Medical Sciences. Since the fall of 2017, Shiraz University of Medical Sciences has started working on this new educational model as an innovation in the midwifery educational structure to promote the quality of midwifery clinical education, provide better clinical services, and pave the ground for ensuring reproductive health (Table 1).

**Table 1:** Objectives of midwifery clinical faculty model

*Objectives*
According to the Iranian Ministry of Health and Medical Education, the goal of clinical education is the professional empowerment of midwifery students. In recent decades, however, the midwifery graduates’ clinical skills have declined due to a shortage of clinical teachers and the problems in clinical settings. In the MCFM, two midwifery students and a midwifery teacher attend maternity wards for teaching professional skills. The teachers continuously monitor clinical procedures and childbirth, and directly provide clinical teaching to individual students. The continuous presence of clinical teachers in MCFM provides a unique opportunity for scientific acquisition of clinical skills, reduces students’ anxiety, and enhances inexperienced students’ adaptation to clinical settings.

MCFM: midwifery clinical faculty model.

In the first three years of the midwifery Bachelor’s degree program, students receive theoretical teaching and the preliminaries of midwifery skills. In the fourth year, to attain skills in performing natural birth and providing pregnancy care, they start the last year of the midwifery education program. The last midwifery educational stage comprises the internship period and reaching the target childbirth count. In MCFM, last-year midwifery students (7th and 8th semesters) who have successfully passed all their theoretical courses in the first three years, enroll in this educational program to pass their field internship and reach the target childbirth count. In the MCFM, students receive the essential midwifery trainings in two clinical semesters in groups of two with a midwifery teacher. The students are in charge of forming groups of two, which is coordinated with the head of the midwifery department, and midwifery teachers who possess up-to-date knowledge and skills attend the maternity ward on rotating shifts 24 hours a day (on morning, afternoon, and night shifts). The midwifery teachers’ presence and rotating shifts are monthly scheduled by the head of the midwifery department. In the MCFM, midwifery teachers attend the maternity ward on rotating shifts 24 hours a day, and practically teach all midwifery cares to groups of two. This education includes introducing the department, history taking, pregnant women’s admission, and care during labor, monitoring and examination of pregnant women, performing natural childbirth, episiotomy, midwifery emergency management, measures to be taken during midwifery emergencies, and recording patient files. Students specifically focus on learning the clinical principles of midwifery, and acquire the stages of admission and natural birth with the presence of a teacher. The students are in charge of providing care during labor to low- and high-risk mothers, performing labor in low-risk groups, and aiding labor in high-risk groups with the continuous supervision of the clinical teacher. They are also in charge of continuing care for mothers and infants in the fourth stage of childbirth, including the recording of vital signs, providing care for the infants, and teaching breastfeeding to mothers. At the end of a shift, the students should deliver patient reports to the students of the next shift. The teachers in the MCFM should directly supervise the activities of the two students and take responsibility for all their activities. At the end of their work shift, the students should register the reports of the activities in the logbooks and have the logbooks signed by their teachers.

When the clinical course starts, two students and their teacher daily attend the ward based on the schedule sent by the head of the department to the clinical ward. On the first day, the teacher familiarizes the students with the rules and regulations and their responsibilities, and the students observe care provision by their teacher during labor and childbirth. During the first days of the clinical practice, the students are prepared by their teacher for providing clinical care to pregnant women during labor, and for beginning their practice in the maternity ward. The teacher educates patient visit stages, history taking, and admission to inexperienced students individually. In the next stages, each student should visit three to four patients and, when the visits and history taking are done, he/she should briefly report the history and the results of the examination to the teacher. The teacher fully supervises the history taking and examinations done by the student (Table 2). After admitting the patient to the maternity ward, the student should provide all the care services, including monitoring, fetal heart check, IV injections, inductions, and examinations, and draw up the delivery chart. The teacher should supervise the accurate implementation of these stages. The transfer of the pregnant woman to the delivery room is done by the student under the supervision of the teacher. The stages of natural delivery (prep and drape, episiotomy, and childbirth) are performed by the first student, aided by the second student, and supervised by the teacher. One hour after the delivery, all care is provided including the mother’s checkups, infant’s physical exam, breastfeeding training, and postpartum care by the first student and supervised by the teacher. In the MCFM, both students participate in diagnostic and therapeutic decision-making under the supervision of their teacher. At the end of the working shift, the teacher reviews and signs the notes and reports made by the students. At this time, the report of the ward and the group’s performance is also presented to the next group by the teacher and the students.

**Table 2:** Ground rules of midwifery clinical faculty model

*Ground rules*
The students attend the ward on scheduled shifts to attain professional skills and perform multiple visits (history taking and examinations) under the direct supervision of the teacher. The students should present a brief report of the history and visit plan based on the information obtained from the examination and the patients’ medical records. At the beginning of every shift, the teacher selects some patients to be examined and cared for by the students based on the patients’ history and health status. The students provide all care during labor, including care for the mother and fetus, childbirth, and postpartum care, under the supervision of the teacher. At the end of the shift, the students attend a meeting to review the activities, complete the documents on daily evaluations, and have the documents signed by the teacher.

The important point in this model is that each pregnant woman receives care and services during admission from only one student. This model does not affect the scheduling patterns or the length of the visit period at the clinic and the admission at the maternity ward. The students should provide care related to natural and physiological childbirth to mothers in the labor ward. However, if any emergency or high-risk case is encountered during admission and labor, it is examined by the teacher and, if necessary, reported to the obstetrician-gynecologist for further examination. Finally, the midwifery teacher decides whether the students should continue providing care to pregnant women, or more specialized care should be provided by the medical team.

The MCFM provides opportunities for the continuous presence of midwifery teachers in clinical settings, and promotes the continuous education and monitoring of the students during the clinical course, thereby enhancing interactions and bringing about deeper learning for the students. This model promises an innovative strategy for responding to the needs of midwifery education and achieving sustainable development goals to ensure the health of pregnant women and promote the health of society. Recruiting competent professors, enhancing the cooperation between teachers and personnel, improving the atmosphere of the educational setting, fortifying the equipment and facilities, making theoretical and clinical topics compatible, and developing midwifery clinical guidelines can help promote the quality of this educational program.

## Data Availability

Data sharing is not applicable to this article as no new data were created.
